# Electric Field Based Therapies in Cancer Treatment

**DOI:** 10.3390/cancers12113420

**Published:** 2020-11-18

**Authors:** Marie-Pierre Rols, Muriel Golzio, Jelena Kolosnjaj-Tabi

**Affiliations:** Institute of Pharmacology and Structural Biology, 205 Route de Narbonne, 31400 Toulouse, France

Enormous progress has been made in pulsed electric field-based therapies since J. Teissié reported the occurrence of electric field-induced transient pores in phospholipid bilayer vesicles in 1981 [1]. The transient pores, which occurred upon application of short (microsecond) external electric field pulses were attributed to the dielectric breakdown of the bilayer structure and did not damage the lipid membrane [1]. While the term “electroporation” (also known as electropermeabilization or the formation of transient permeant structures) took some time to anchor within the vocabulary of the entire scientific community, experiments showed that pulsed electric field could have numerous biological consequences. Among them, the induction of ATP synthesis [2], onset of cell fusion [3], generation of cell hybrids [4], induction of cytoskeletal reorganization [5], transfection of cells [6,7,8], delivery of molecules including cytotoxic drugs to cells [9], and anticancer therapy [10] set the bases for therapeutic applications.

This Special Issue covers a number of hot topics in the field of electric field based therapies in cancer treatment. Original research and a review article present recent advances in calcium electroporation [11,12,13,14], the potential of expandable electrodes is presented in a porcine model undergoing open body surgery, laparoscopy and endoscopy [15], and the extension of the time of blood brain barrier disruption is highlighted after application of high frequency electroporation [16]. In addition to a review providing the state of the art in cytoskeletal alterations after electroporation [17], original research articles also present the induction of immunogenic cell death by nanosecond pulsed electric fields [18] and describe protocols for the optimization of DNA electrotransfer [19,20].

In addition to electrochemotherapy and irreversible electroporation, that already proved their efficacy to treat cancer, the combination of calcium ions with high intensity electric field pulses is emerging in clinics. As calcium ions are implicated in cell death regulation, the amplification of calcium ions uptake upon electropermeabilization results in acute and severe ATP depletion associated with cancer cell death. This approach has been used in clinics and the treatment modality is thoroughly reviewed by Frandsen et al. [14]. In addition to this comprehensive review, Agoston et al. [12] presented a Phase II Clinical trial (NCT03628417) where they compared the efficacy of calcium-based electroporation with bleomycin-based electrochemotherapy. The two approaches lead to a similar tumor response but adverse reactions, such as ulceration and hyperpigmentation, were less common after calcium-based electroporation [12]. Moreover, as highlighted by Gibot et al. [13], calcium electroporation is not genotoxic. In addition to electrochemotherapy protocols, calcium ions were shown to delay tumor growth upon combination with irreversible electroporation, as reported by Novickij et al. [11]. Their protocol was not only efficient against primary tumors in a murine model, but also destroyed the tumor microenvironment and induced anti-tumor immune response. A similar phenomenon of immunostimulation was also observed by Rossi et al. [18], who applied nanosecond pulsed electric fields in murine cancer models.

Significant advances were made in electric field-based delivery of DNA [21] to cancer cells. Indeed, protocols still need to be optimized. In this context, Sieni et al. [19] described a useful three-dimensional cellular scaffold, which is rich in extracellular matrix and appears particularly attractive for gene electro transfer studies. As electrotransfer may decrease cell viability, Wang et al. [20] show that inhibition of caspases post electrotransfer may significantly increase cell viability, without compromising the T cell receptor disruption efficiency.

High frequency electroporation was efficiently used to transiently disrupt the blood brain barrier in vivo in a healthy rat brain model, as shown by Lorenzo et al. [16]. In the mentioned study, the blood brain barrier could remain focally disrupted for 72 h following the application of high frequency electroporation, and returned to its normal 96 h following pulse exposures. This finding thus suggests a useful approach to permeate the blood brain barrier and promote drug diffusion into brain parenchyma. 

Last but not least, this special issue also includes a review by Graybill et al. [17] describing cytoskeletal disruption after cellular exposure to pulsed electric fields. This extensive review summarizes nearly 200 studies describing cytoskeletal disruption [22] englobing Teissié’s pioneering works [23] and a series of cutting-edge papers detailing the mechanisms and outcomes of cytoskeletal disruption.

We hope that this Special Issue will be of interest to a vast number of researchers and that it will encourage new ideas and scientific discoveries. The editors are highly grateful to the editor in chief, editorial staff, reviewers, and to all contributors. We look forward to meeting you again at the forthcoming 4th World Congress in September 2021 in Copenhagen, Denmark.
